# Transformative Learning in Digital Bioethics Education: An Interdisciplinary Lecture Series on Human–Animal Relationships

**DOI:** 10.3390/ani15233398

**Published:** 2025-11-25

**Authors:** Kim Widmann, Theresa Sophie Busse, Jan P. Ehlers, Julia Nitsche

**Affiliations:** 1 Department of Didactics and Educational Research in Health Science, Faculty of Health, Witten/Herdecke University, 58455 Witten, Germany; kim.widmann@uni-wh.de (K.W.); jan.ehlers@uni-wh.de (J.P.E.); 2 Digital Health, Faculty of Health, Witten/Herdecke University, 58455 Witten, Germany; theresa.busse@uni-wh.de

**Keywords:** human–animal relationships, transformative learning, bioethics in health professions, interdisciplinary teaching, planetary health, one health education

## Abstract

This study presents an interdisciplinary digital lecture series on human–animal relationships developed at Witten/Herdecke University. The course explored ethical, ecological, and health-related aspects of animal–human interactions within the frameworks of One Health and planetary health. Grounded in Transformative Learning Theory, the 14-week series combined expert-led lectures with live polls, Q&A sessions, and a participatory Barcamp. The course was open to students and the general public. Quantitative data were collected on participation, interaction, and reflective journal submissions. A total of 1382 individuals registered while the mean number of participants per session was 470.5 (SD = 60.9). Sessions on ethically sensitive topics such as animal ethics, hunting or transportation generated particularly high engagement during sessions. In total, 291 journals were submitted, demonstrating reflective depth and personal involvement. The findings underscore the potential of digital lecture series to foster ethical awareness and interdisciplinary dialogue. The model offers a transferable approach for integrating animal ethics into health curricula and advancing bioethical literacy across audiences. This manuscript presents Part 1 of a two-part study focusing on implementation, reach, and engagement; Part 2 will report pre–post survey outcomes using validated instruments to assess learning and perspective change.

## Simple Summary

Human–animal relationships are central to society but often raise unresolved ethical, ecological, and health-related questions. These topics remain underrepresented in higher education, despite their relevance for sustainable healthcare and public debate. To address this gap, Witten/Herdecke University developed an interdisciplinary digital lecture series open to students and the general public. The series combined expert lectures with interactive elements, including polls, Q&A sessions, and a participatory Barcamp (a participant-driven “unconference” in which attendees co-create the agenda and work in small, self-selected sessions). 1382 individuals registered, and ethically sensitive themes elicited particularly high engagement. The mean number of participants per session was 470.5 (SD = 60.9). Nearly 300 reflective journals were submitted, documenting critical self-reflection and perspective shifts among participants. The findings highlight the potential of digital formats to reach large, diverse audiences and to foster interdisciplinary dialogue and transformative learning. This model provides a transferable approach for integrating animal ethics and human–animal relationship topics into health and higher education curricula, thereby strengthening ethical awareness and planetary health literacy in both academic and societal contexts. This manuscript presents Part 1 of a two-part study focusing on implementation, reach, and engagement; Part 2 will report pre–post survey outcomes using validated instruments to assess learning and perspective change.

## 1. Introduction

### 1.1. Background

In an era of biodiversity loss and significant planetary boundary overshoot [1] fostering a new social consciousness about our treatment of nature and the environment is paramount. However, individual disciplines often only address this challenge from a single perspective. For example, veterinary medicine primarily deals with animals, human medicine with humans, and the social sciences with society. The One Health [2] and Planetary Health approaches [3] describe a transdisciplinary framework that combines these perspectives to find shared solutions. Similarly, Human–Animal Studies [4] examine the relationship between humans and animals from ethical, medical, cultural, and legal viewpoints, contributing to a more integrated coexistence between humans and other species in society.

Higher education and informal learning both play a pivotal role in shaping societal understanding of health, human–animal relationships, and bioethics, and can thereby advance One Health and Planetary Health goals [5]. However, higher education remains largely organized along disciplinary boundaries. Specialized degree programmes often focus on expertise in one domain, while future challenges require transdisciplinary competencies that foster ethical reflection and collaboration across fields [6]. Integrating approaches such as Human–Animal Studies and One Health education into academic curricula may therefore be key to cultivating such future skills and conveying the value of non-human life to students from diverse disciplines.

Building on this broader perspective, the present study situates itself within the growing field of Planetary Health and One Health education. Human health is inextricably linked to the social and ecological conditions in which people live. Against this backdrop, the One Health approach—introduced more prominently into the global health discourse in the early 2000s [7]—has gained increasing relevance. This concept recognizes the interconnectedness of human, animal, and ecosystem health [8] and provides a framework for developing multidisciplinary responses to complex global health challenges.

Humans and animals coexist in diverse and mutually dependent relationships—whether in the context of companion or farm animals, within urban ecosystems, or in biomedical research. These relationships are often characterized by a striking ambivalence: while strong emotional bonds frequently develop between humans and their pets, forms of systematic exploitation—such as industrial livestock farming or invasive animal experimentation—remain widely accepted by society. For instance, public awareness of the roles of farm animals in food systems and research animals in biomedical innovation remains limited [9,10]. From both an ethical and public health perspective, this discrepancy is troubling and can be explained only in part by anthropocentric attitudes or speciesism.

Industrial animal agriculture poses significant risks to human health, particularly due to its role in the emergence of zoonotic diseases. Moreover, intensive livestock production is a major contributor to the climate crisis, which in turn leads to extreme weather events, rising global temperatures, and altered precipitation patterns, all of which have both direct and indirect consequences for human health [11,12]. Heatwaves increase mortality rates [13], while shifting climatic conditions facilitate the spread of infectious diseases [14]. The climate crisis also threatens agricultural productivity, especially in less technologically advanced regions, where it contributes to growing insecurity in food production and availability [15]. In addition, the climate-driven loss of biodiversity and environmental pollution are placing increasing strain on global health systems [16]. These climatic stressors also adversely affect non-human animals through heat stress, habitat alteration, and disease dynamics, further linking animal and human health [17]. These developments urgently call for a transformation in dietary habits, agricultural policy, and the design of resilient healthcare systems.

The human–animal relationship also raises critical ethical and scientific questions in the field of medical research. Despite growing criticism, animal experimentation remains widespread, with concerns related not only to ethical justification, but also to scientific validity and translational applicability. While invasive experimentation compromises animal welfare, it is often justified on anthropocentric grounds of expected biomedical benefit, regulatory requirements, and perceived translational value [18]. Ethical review frameworks typically operationalize this through human-centred harm–benefit analyses that weigh anticipated gains for human health against the pain, distress, and death imposed on animal subjects, a rationale increasingly criticized for downplaying animals’ moral status and the limits of translational inference.

Beyond its relevance for human and planetary health, the topic of human–animal relationships also provides valuable impulses for animal science, ethics, and welfare research. These fields are inherently linked to the ethical and societal challenges of human–animal interactions, yet educational perspectives have often been overlooked. Innovative teaching approaches that unite scientific expertise with ethical reflection can advance these disciplines and strengthen professional competencies [19,20]. Such approaches offer veterinarians and animal scientists new ways to integrate animal ethics and welfare perspectives into education and practice, thereby fostering ethical awareness and interdisciplinary collaboration.

Despite the clear relevance of animal ethics, ecological issues, and veterinary topics for both society and health professions, these areas are still largely underrepresented in healthcare education. A shift in training paradigms is needed to adequately prepare future professionals [21]. At the same time, significant knowledge gaps and a lack of public engagement with these topics persist. To foster informed and future-oriented decision-making in the context of human–animal relations, integrative educational formats are essential. Given the wide-ranging interconnections described above, it is crucial to design educational formats in an interdisciplinary manner and to provide adequate space for addressing the complexity of these issues. University-level teaching plays an increasingly important role in fostering interdisciplinary collaboration and deepening the understanding of the intricate relationships between humans, animals, and the environment [22]. One Health topics require lecturer from diverse backgrounds to share their knowledge and experience to educate students across disciplines—thereby laying the foundation for transdisciplinary communication and collaboration from the outset [23]. In this context, health professionals are trusted messengers and change agents in One Health and Planetary Health initiatives [24]. Engaging this group is crucial for translating knowledge into practice across clinical, public health, and educational settings [25].

Villanueva-Cabeza et al. further emphasize that One Health education should extend beyond medical and veterinary students and is most effective when taught to diverse student cohorts [26]. Promoting planetary, holistic, and cross-professional thinking requires input from a broad range of academic domains, including natural sciences, technology, engineering, mathematics, and medicine, as well as the humanities, arts, and social sciences. These perspectives can contribute significantly to a more comprehensive understanding of the multifaceted relationship between humans, animals, and the environment. Health-related degree programs have jointly committed to education for sustainable healthcare [27], with a particular focus on advancing planetary health literacy [28].

To engage such a transdisciplinary audience, digital lecture series provide an ideal format. One of the major strengths of the online model lies in its flexibility to cover a wide range of interdisciplinary topics through rotating guest lecturers. This approach allows for the development of a nuanced and multi-perspective view of the human–animal–environment relationship, while also helping to sustain participants’ motivation over time [29].

Moreover, this format enables a high degree of participant interaction. In contrast to traditional lectures that often focus solely on knowledge transmission, digital lecture series that include interactive elements—such as live polls and moderated discussions—encourage learners not only to absorb information but also to critically engage with it and place it in a broader societal context [30]. Interactive and enthusiastically delivered teaching formats have been shown to be more effective for long-term learning and motivation, as they stimulate active engagement with content and support the development of problem-solving skills [31,32].

Another important factor is the size and diversity of the audience. While seminars and smaller lectures are often limited in participant numbers, a digitally delivered lecture series can reach a much broader public. This also makes it possible to include participants from multiple universities and from the general population, regardless of geographic location—an essential feature when the goal is to promote widespread societal engagement and action [33,34].

### 1.2. The Lecture Series “Human–Animal Relationship”

#### 1.2.1. Studium Fundamentale

At Witten/Herdecke University (UW/H), the development of personal competencies is considered a central component of academic education. To support this aim, every degree program includes a dedicated component called the Studium Fundamentale (fundamental studies). As part of this format, students from all faculties, including the Faculty of Health (encompassing Human Medicine, Nursing Science, Dentistry, and Psychology) as well as the Faculty of Management and Society, select courses from the areas of reflection, communication, and culture to fulfill approximately 10% of their total course requirements. This enables them to engage in interdisciplinary learning and personal development alongside their professional studies [35]. These courses bring together students from across faculties, disciplines, and cohorts in shared learning environments.

#### 1.2.2. Seminar “Human–Animal Relationship” as Initial Offering

The Studium Fundamentale (fundamental studies) provides space for exploring issues at the interface of human–animal relationships and climate change. In the summer semester of 2021 and the winter semester of 2021/2022, the seminar “Human–Animal Relationship” was offered for the first time at UW/H. With a limit of 30 participants, the course was designed to explore the subject not only from a medical or veterinary perspective but also by addressing ethical and economic dimensions. In weekly sessions, student groups independently selected a topic relating to human–animal relationships, presented it to the course, and facilitated a group discussion. Based on the student contributions from the winter term 2021/2022, a collaborative publication was produced [36].

Building on this success, the idea emerged to scale the concept—maintaining the relevance of the subject matter while expanding the format and audience. Thus, in the winter semester of 2024/2025, the course was restructured and offered as a university-wide digital lecture series within the framework of the Studium Fundamentale (fundamental studies).

#### 1.2.3. Format and Content of the Lecture Series

**Figure 1:**
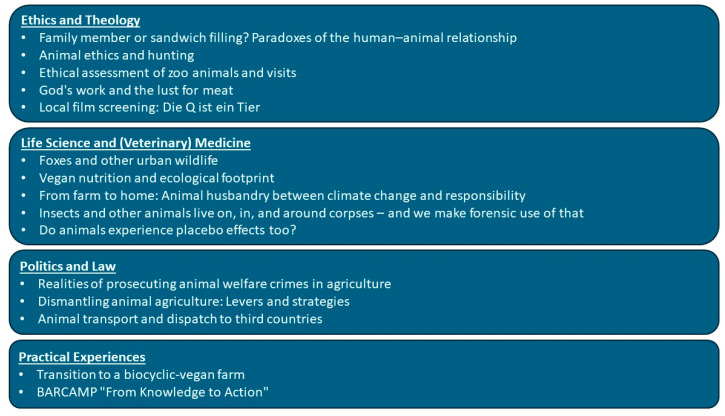
Thematic structure of the lecture series’ modules.

The weekly sessions ran over a four-month period (October 2024–January 2025) and were moderated by three lecturers (JE, JN, TSB) from UW/H. Each session lasted 90 min and consisted of a 60 min presentation by invited speakers from various disciplines related to the overarching theme of human–animal relationships (see Table 1). Figure 1 provides an overview of the thematic structure of the lecture series, summarizing the individual modules according to their disciplinary focus areas in ethics and theology, life sciences and (veterinary) medicine, politics and law, and practical experiences. The subsequent 30 min open discussion was designed to encourage the development of forward-thinking visions and to support participants in forming their own ethical and professional stances.

To encourage interaction, live polls, surveys, and chat-based engagement were integrated into both the presentations and discussions. At the end of the series, a dedicated session was conducted in the format of a participatory Barcamp workshop, aiming to translate knowledge into concrete action (“From Knowledge to Action”). A Barcamp is a form of unconference, i.e., a participant-driven, collaboratively organized event where attendees co-create the agenda and engage in small-group sessions on self-selected topics—an approach shown to promote diversity of perspectives and high participant satisfaction and engagement in educational settings [37]. This session consisted of eleven thematic discussion rounds, each introduced by a brief impulse talk, followed by small-group work in which participants collaboratively developed ideas for applying what they had learned (see Table 2).

The platform Zoom (Zoom Video Communications, Inc., 55 Almaden Blvd, 6th Floor, San Jose, CA 95113, USA) was used for both the lecture series (via webinar mode) and the Barcamp (via meeting mode), ensuring broad accessibility. Participants were asked to register for the event in advance to receive a personalized link, which also allowed for attendance tracking. A supplementary in-person event—a film screening of “Die Q ist ein Tier”—was offered at multiple locations to foster emotional engagement with the topic and provide opportunities for informal networking among participants. This film by director Tobias Schönenberg and screenwriter Hilal Sezgin aims to offer a humorous take on the topic of animal slaughter by following a criminal case involving slaughterhouse waste, highlighting the absurdity of eating animals [38]. The locations and dates of the film screenings can be seen in Table 3. In a final interactive quiz session using Kahoot, participants were able to test and reflect on their acquired knowledge in a playful, self-directed manner.

#### 1.2.4. Course Objectives

The lecture series “Human–Animal Relationship”, offered in the winter semester 2024/2025, was designed as an interdisciplinary teaching format. The aim was to develop and evaluate an innovative, participatory educational program focusing on the diverse dimensions of human–animal relationships. The central question was whether a public university-wide lecture series could serve as an effective format to raise awareness among students and external participants and to provide a platform for interdisciplinary dialogue. Three main objectives guided the course:

- Knowledge transfer: To promote a comprehensive understanding of human–animal relationships, including ethical, legal, ecological, and health-related dimensions.
- Interdisciplinary exchange: To facilitate cross-disciplinary dialogue among students from medicine, psychology, and economics at UW/H, as well as external students from diverse academic fields, interested members of the public, professionals, and volunteers.
- Community building and activation: To encourage personal reflection, civic engagement, and intrinsic motivation with the goal of transforming knowledge into action.

#### 1.2.5. Research Questions and Hypotheses

In addition to its didactic and thematic focus, the lecture series was accompanied by a scientific evaluation to assess its effectiveness in terms of knowledge acquisition and shifts in perspective. This paper focuses on the following research questions:

- How can a lecture series on human–animal relationships attract and engage a broad audience?

**Hypotheses** **1.** *The lecture series will attract a large audience, reflected in high registration and attendance numbers across sessions.*

- To what extent can professionals in the healthcare sector—that are not primarily linked to animals—be motivated or engaged with such topics?

**Hypotheses** **2.** *Individuals from health-related fields will participate actively in the lecture series and engage with human–animal relationship topics, as reflected in observable interaction measures.*

This article presents the first part of a larger research project. The focus of this article lies on the implementation, reach, and acceptance of the lecture series. We report descriptive findings regarding participation, interaction, and course design in order to evaluate whether such a format can successfully engage a broad and diverse audience. A second article will follow, based on a pre- and post-course survey, which will provide a more rigorous evaluation of transformative learning outcomes and changes in participants’ perspectives.

The second paper will focus on the following research questions:

- To what extent do participants, prior to the course, perceive human–animal relationships and One Health as relevant to human, animal, and planetary health?
- In what ways do attitudes differ across various participant groups?
- How does participation in the lecture series “Human–Animal Relationship” in the winter semester 2024/2025 influence participants’ perspectives?

With this two-step design, we aim to offer both a systematic account of the course implementation and, in a subsequent publication, an empirical assessment of its educational impact.

## 2. Materials and Methods

**Figure 2:**
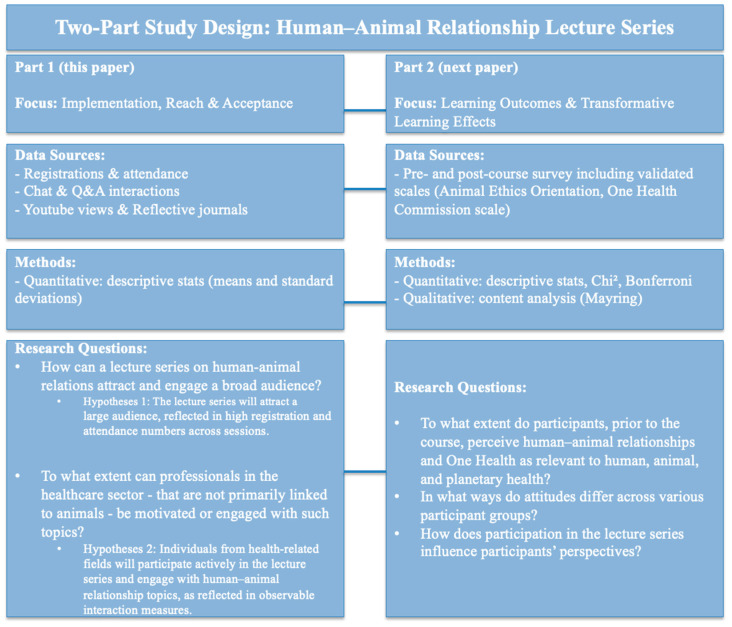
Methodology overview of the Two-Part Study Design.

Ethical approval for this study was granted by the ethics committee of UW/H (reference number 186/2024). The following methodology was applied to address the research questions. As outlined above, this project is a two-part study. While Part 1 (this paper) reports on implementation, reach, and acceptance, Part 2 will present pre–post survey results on participants’ learning outcomes. Figure 2 summarizes the overall study design.

### 2.1. Theoretical Framework: Transformative Learning Theory

The lecture series is conceptually grounded in Mezirow’s Transformative Learning Theory, which posits that deep learning occurs through critical reflection on previously held assumptions [39]. This process is often initiated by ethically or emotionally challenging content and fosters lasting shifts in perspective and behavior [40]. Interdisciplinary and values-based formats—such as those combining sustainability and social justice topics—create a “third space” in which diverse perspectives meet, enabling reflective discourse and co-constructed knowledge that effectively trigger transformative learning outcomes [41,42].

### 2.2. Participants

The course was open to both students at UW/H and external participants, including students from other universities and members of the public. This open-access structure broadened the range of perspectives and facilitated wider societal engagement. The diversity shown in both the most common study programs and locations of the participants, as illustrated in Table 4 and Table 5, emphasize how the series attracted attendees from many academic backgrounds and locations. External students were able to request certification of participation from the instructors and seek academic credit at their home institutions. No restrictions were placed on the participants’ field of study or academic level.

### 2.3. Data Collection

The evaluation of the lecture series followed a mixed-methods approach, incorporating both quantitative and qualitative data collection strategies. This paper focuses specifically on the quantitative findings.

Participant engagement was continuously documented through the total number of registrations and weekly attendance records.

A key element of data collection involved the analysis of interaction metrics within the Zoom platform. These included the number of questions submitted via the Q&A (questions and answers) function, as well as chat messages exchanged during discussions—both between participants and in communication with speakers and facilitators. In addition, the number of live polls conducted during each session and the corresponding number of responses were recorded as indicators of active participation.

Public engagement with the lecture series was also assessed via YouTube analytics, capturing the number of views of recorded sessions.

As part of course requirements, some participants submitted reflective learning journals that documented their personal insights and critical reflections over the course of the series each in relation to a specific lecture. Reflective journals were optional for attendees and required additional time after sessions, which likely contributed to lower submission rates relative to live interaction metrics. These served as a qualitative complement to the quantitative analysis, offering insight into individual learning processes and participant perceptions.

### 2.4. Data Analysis

Participant data were exported from Zoom and initially sorted and cleaned in Excel (e.g., removal of duplicate entries). The cleaned dataset was then imported into SPSS (Version 29.0, IBM Corporation, 233 S. Wacker Drive, 11th Floor, Chicago, IL 60606-6307, USA), where descriptive statistical analyses were conducted to summarize central participation and engagement metrics. Participation reach and engagement were assessed by examining both cumulative and session-specific attendance data.

Interaction rates—including chat activity, Q&A submissions, poll participation, and attendance at the Barcamp workshop—were analyzed quantitatively to evaluate levels of active involvement.

In addition, the number of YouTube views and submitted learning journals were considered as complementary indicators of content engagement and personal reflection.

This multi-layered analysis aimed to assess the extent to which the lecture series functioned as an effective educational format for One Health, and to identify the key factors that supported interactive participation and meaningful content engagement. The findings are intended to inform future development of interdisciplinary teaching models and to contribute to the design of innovative educational formats in the field of human–animal relations and One Health.

## 3. Results

### 3.1. Participation and Registration

A total of 1382 individuals registered for the “Human–Animal Relationship” lecture series during the winter semester 2024/2025.

An overview of attendance per session is presented in Table 6. The mean number of participants per session was 470.5 (SD = 60.9). The highest attendance was recorded on 7 November 2024 (548 participants), followed by 17 October and 24 October 2024 (each with 529 participants). Lowest attendance was noted on 23 January 2025 (299 participants) and 30 January 2025 (399 participants).

### 3.2. Interaction Data: Chat and Q&A Messages

To assess participant interactivity, both chat messages among attendees and Q&A submissions directed to the speakers were recorded. The mean number of chat messages per session was 210.1 (SD = 168.1). Notably high levels of chat activity were observed at the beginning of the lecture series; for example, 811 chat messages were recorded on 10 October 2024. In the later stages, participation declined—only 71 messages were registered on 12 December 2024. The mean number of Q&A messages submitted to lecturers per session was 13.2 (SD = 7.1), with the highest number recorded on 7 November 2024 (29 messages) and the lowest on 16 January 2025 (3 messages).

### 3.3. Polls Conducted During the Lecture Series

Live polls were conducted during selected sessions throughout the lecture series. In total, polls were held on eight occasions. The mean number of polls per lecture was 3.0 (SD = 2.1), and the average number of responses per session was 724.1 (SD = 578.1). Particularly high levels of participation were noted on 10 October 2024 (1515 responses across five polls) and 31 October 2024 (1644 responses across six polls). In contrast, sessions with lower participation included 14 November 2024 (242 responses to one poll) and 21 November 2024 (246 responses to one poll).

### 3.4. YouTube Views and Submitted Learning Journals

Each lecture session was recorded and made available to the public via YouTube (see Table 7). The average number of views per recording was 315.3 (SD = 162.19). The highest number of views was recorded for the first session on 10 October 2024 (676 views), while the lowest occurred on 5 December 2024 (131 views).

In addition, a total of 291 reflective learning journals were submitted by participants as part of their coursework (see Table 7). Of these, 31 journals were general motivation statements or introductory reflections. The highest number of journals was submitted for the introductory session on 10 October 2024 (32 submissions). The lowest submission numbers were for the Barcamp workshop on 23 January 2025 (2 journals) and the session on 12 December 2024 (6 journals).

### 3.5. Certificates of Participation and Performance

**Figure 3:**
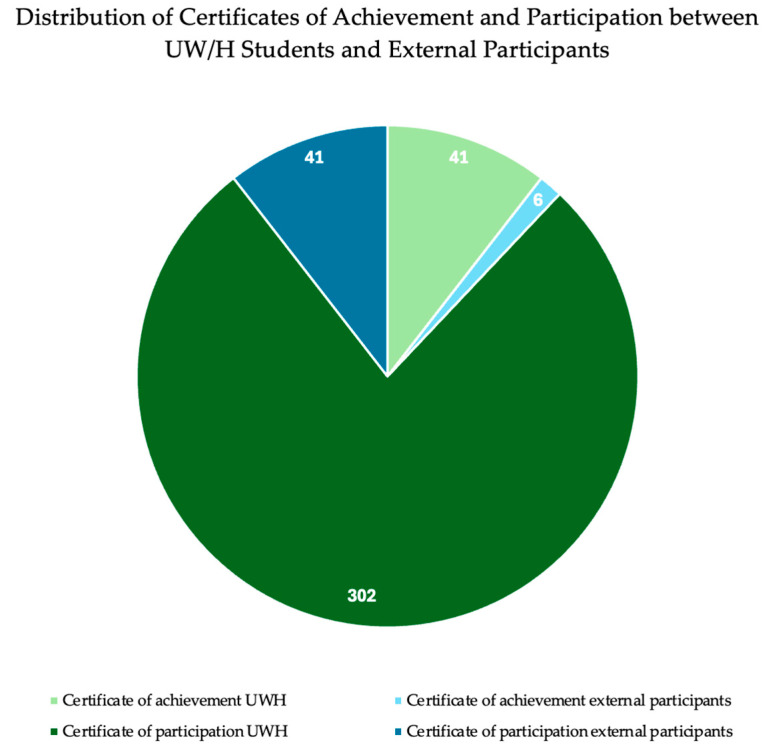
Distribution of Certificates of Achievement and Participation between UW/H Students and External Participants.

Participants had the opportunity to receive either a certificate of participation or a certificate of achievement for their involvement in the lecture series. A certificate of participation was issued for documented attendance of 12 lecture sessions, while a certificate of achievements required completion of additional four reflective learning journals. This option was available to both internal students at UW/H and external attendees (see Figure 3). Of the 443 registered UW/H students, 302 (68.1%) received a certificate of participation, and 41 (9.3%) were issued a certificate of achievement. Among the external participants, 41 individuals received a participation certificate and 6 received a certificate of achievement, all of which were students.

## 4. Discussion

This manuscript reports on the development and implementation of the lecture series “Human–Animal Relationship”, offered at UW/H as an educational format open to both internal students and external participants, including students from other universities and members of the general public. Organized and moderated by three faculty members, the series spanned four months and featured weekly expert-led lectures, a Barcamp workshop, and a film screening. The event was conducted digitally via Zoom and accompanied by a scientific evaluation aimed at assessing its effectiveness in terms of knowledge acquisition and changes in participant perspectives. A mixed-methods approach was used to collect a wide range of data. In this manuscript, the focus was put on the quantitative findings, including participation numbers and registrations, interaction behaviour (chat and Q&A messages), live poll engagement, YouTube view counts, submitted learning journals, and issued certificates of achievement or participation.

The high number of registrations and consistently strong attendance throughout the semester highlight the substantial public interest in the topic of human–animal relationships. YouTube view counts further suggest sustained interest beyond the live events, as well as potential sharing or recommendation of the recordings. Interaction rates—particularly chat and Q&A messages and poll responses—were strikingly high in the first few sessions, indicating initial enthusiasm and the effectiveness of interactive, activating elements in digital teaching formats [43]. Over time, however, a gradual decline in interaction was observed, which may reflect the onset of “video conferencing fatigue” [44,45]. Video conferencing fatigue refers to the cumulative cognitive load inherent to synchronous online communication, arising from intensified self-presentation demands, limited nonverbal feedback, and sustained attentional effort [46,47]. Evidence suggests that fatigue can be mitigated through interface and format adjustments—such as disabling self-view, fostering active participation, and optimizing visual settings [45]—as well as through practical measures including micro-breaks, shorter meeting segments, and alternating synchronous and asynchronous tasks [48]. Content analysis further suggests that emotionally and ethically charged topics such as animal ethics, hunting, or animal transport generated particularly strong participant engagement. The higher number of learning journals submitted in response to these topics also points to more intense individual reflection.

The findings demonstrate that the lecture series served as an accessible and effective format for communicating complex and transdisciplinary topics such as the human–animal relationship. The combination of expert lectures, emotionally engaging elements (e.g., film screening), peer contributions, and the concluding Barcamp appeared to strengthen participants’ identification with the content and fostered active engagement [49]. Furthermore, transformative learning formats emphasize the importance of fostering group responsibility, individual agency, and the interplay between critical reflection and affective learning. These approaches support the development of personal and social awareness, which in turn enhances the relevance and depth of course content [50,51]. The lecture series challenged participants across multiple dimensions—emotional, ethical, and academic—and thus created meaningful opportunities for transformative learning.

The lecture series contributed to a broader reflection on contemporary human–animal relationships. Its interdisciplinary approach demonstrated how our understanding of human–animal-relationships is shaped not only by biological and ecological knowledge, but also by cultural narratives, moral frameworks, and sociopolitical contexts. Within the paradigms of “One Health” and “Planetary Health,” animals appear as cohabitants, sentient beings, and indicators of environmental change. The pluralistic content of the lecture series challenges anthropocentric assumptions and invites participants to reconsider their relational responsibility toward nonhuman life. To investigate this further, a pre–post survey on the attendees’ views on human–animal-relationships was conducted, which will be presented in a subsequent paper.

Empirical studies on education about human–animal relations support this approach. Hawkins et al. [52] demonstrate that educational formats can foster more positive attitudes toward human–animal relationships. This aligns with the patterns observed in the present lecture series. The high participation numbers and intensive interaction indicate increased engagement and reflective involvement, which may be associated with shifts in perspective. This connects to the argument made by Feltz et al. [53], who show that educational interventions can increase knowledge about animals in food production and reduce speciesist justifications, even if such changes do not immediately translate into behavioral modification. The pronounced interaction of participants, especially during ethically and conceptually complex sessions, supports this pattern and suggests that learning processes may manifest primarily through discursive and reflective engagement rather than through immediately observable behavior. As in many studies, including the present one, researchers often rely on indirect indicators of learning, highlighting the need for future study designs that capture attitudinal and behavioral change more systematically and over longer periods of time.

This approach is particularly relevant for conveying other societally important and interdisciplinary topics. The 21st century is marked by complex global challenges that require transdisciplinary solutions [54]. Climate change, for example, was a recurring topic in the lecture series, particularly its link to industrial animal use. Within the health sector, the consequences are already apparent: extreme heat events and food insecurity are placing increased strain on healthcare systems. According to the WHO, an average of 489,000 heat-related deaths per year occurred between 2000 and 2019, with vulnerable groups such as women, older adults, and individuals with chronic illnesses disproportionately affected [55,56]. In parallel, climate change impairs agricultural productivity, nutrient bioavailability, and pest resistance, thereby weakening public health outcomes in the long run [57].

A large proportion of lecture participants were affiliated with health professions—an important group in addressing these challenges, both in their clinical expertise and their role as trusted figures and change agents. Rossa-Roccor et al. [58] advocate for a greater commitment from health professionals to drive political change by framing climate change as a health issue. However, current knowledge about climate-related health impacts remains limited among healthcare workers [59]. This underscores the need for broad, interdisciplinary educational formats, particularly for students in health-related fields, to help shape a more sustainable future and to promote both individual and systemic change. Together, interaction metrics (chat, Q&A, polls, Barcamp) and reflective-journal submissions indicate active engagement with human–animal relationship topics. Where affiliation data were available, we observed substantive participation by individuals from health-related fields, supporting our hypotheses that professionals outside animal-focused disciplines can be engaged by this format.

Another domain that would benefit from such educational formats includes politically charged and health-related issues such as wars, authoritarianism, and the resurgence of nationalism, all of which hinder global cooperation [60]. Access to healthcare is increasingly limited in many parts of the world, while rising inequality, mental health burdens, and fragile living conditions call for adaptable und accessible healthcare systems [61,62]. Climate-induced migration further intensifies these public health challenges [63].

Digital transformation also presents new opportunities for promoting sustainability and interprofessional learning through transformative educational formats [64]. The health professions are especially impacted by these shifts and must be adequately prepared for the increasing digitization of the working world. Digitization holds great potential, for example, in saving resources [65], facilitating interprofessional exchange, and enhancing patient empowerment through improved access to information and communication [66]. At the same time, there is a risk that digital health innovations may exacerbate social inequalities. Individuals with lower income, limited education, or poor digital literacy are less likely to benefit from these developments [67,68].

The demand for interdisciplinary education is therefore broad and multifaceted—essential not only for society but also for preparing future professionals to navigate ethical complexity and real-world challenges. When embedded systematically into curricula, such formats foster improved collaboration, communication skills, and mutual role understanding among learners from different disciplines [69]. The most lasting effects are observed when interdisciplinary learning is integrated longitudinally throughout a program of study, strengthening cross-cutting competencies and building a foundation for effective cooperation in complex professional settings [70,71].

### 4.1. Limitations

Despite the positive reception of the lecture series, several methodological and structural limitations must be considered. The interactive tools used during the course—such as live polls and the Barcamp workshop—were not systematically assessed in terms of their specific didactic impact; future iterations of the lecture series would benefit from a more nuanced analysis of their usage and educational effectiveness.

Additionally, it should be noted that the particularly high attendance during the first session may be partly attributed to its role as an introductory event, during which organizational details as well as requirements for certificates of achievement and of participation were outlined.

Although a self-selection bias cannot be entirely ruled out due to the voluntary nature of participation, the large sample size and the heterogeneity of participants mitigate this concern. The cohort included individuals motivated by the thematic focus as well as those primarily interested in the digital format, resulting in a balanced mixture of perspectives. In contrast to our earlier small-scale seminar with 30 students, the registration of more than 1300 participants substantially reduces the likelihood that individual predispositions substantially influenced the overall findings.

Technical limitations of the Zoom platform—such as the restricted functionality for group work in webinar mode—should also be taken into account when planning similar formats in the future.

### 4.2. Outlook

The results of the evaluation highlight the strong potential of the Human–Animal Relationship lecture series as an innovative educational model. Integrating similar formats into mandatory components of academic programs, particularly in the context of Planetary Health or One Health, appears beneficial to promote sustainability-oriented and ethically grounded thinking early in students’ education. Beyond curricular integration, the event also offers opportunities to strengthen its citizen science dimension. Research has shown that involving participants in data collection and evaluation not only enhances environmental awareness and scientific literacy but also increases engagement and motivation in academic settings [72]. Greater inclusion of civil society actors—for example through collaborative projects or open-access platforms—could extend the impact of the lecture series beyond academia and contribute to social innovation [73].

Additionally, the internationalization of the format represents a promising next step. Collaborations with other universities, non-governmental institutions, or international institutions could help globalize and diversify the discourse on human–animal relationships. The scalability and repeatability of the lecture series should also be assessed to further its reach and impact.

A planned qualitative and quantitative analysis of questionnaire data collected before and after the course will be presented in a second paper. These findings are expected to provide further insights into participants’ knowledge acquisition and changes in attitudes, forming a foundation for the continued development and didactic refinement of the format.

## 5. Conclusions

The evaluation of this lecture series demonstrates that digital, interdisciplinary formats can effectively address complex questions at the intersection of human, animal, and environmental health. High participation and engagement underline the societal relevance of the topic and the capacity of interactive methods to stimulate reflection and dialogue.

The results show that collaboration between the animal science community and the education community can be highly effective in reaching audiences beyond their own fields. The high participation numbers, broad reach, and intensive interactions highlight how combining subject expertise with innovative teaching approaches enables wider societal engagement and creates meaningful impact outside the immediate academic community.

Embedding such approaches into curricula and extending them internationally may contribute to advancing One Health and planetary health literacy. For healthcare professionals, integrating human–animal relationship content within One Health and Planetary Health curricula can strengthen ethical reasoning, public communication, and advocacy capacities relevant to clinical and community practice. By linking ethical awareness with participatory learning, digital education can prepare professionals and the public to navigate the challenges of human–animal relationships in an increasingly interconnected world.

## Figures and Tables

**Table 1:** Contents of the lecture series in the winter semester of 2024/2025.

Date	Content	Speaker(s)
10.10.24	Introduction: Family member or sandwich filling? Paradoxes of the human–animal relationship *	Dr. Julia Nitsche (Department of Didactics and Educational Research in Health Care), Prof. Dr. Theresa Busse (Junior Professorship for Digital Health), Prof. Dr. Jan Ehlers (Head of Department of Didactics and Educational Research in Health Care)
	Information about exam requirements	
17.10.24	Realities of prosecuting animal welfare crimes in agriculture	Dr. Johanna Hahn, LL.M. (Harvard), FAU Erlangen-Nuremberg (Postdoctoral researcher at the Chair of Criminal Law, Criminal Procedure Law, and Legal Philosophy)
24.10.24	Dismantling animal agriculture: Levers and strategies	Dr. Friederike Schmitz (Author, Faba Konzepte e.V., Berlin)
31.10.24	Transition to a biocyclic-vegan farm	Maik and Marco Möller, Möllers Morgen, Lentföhrden
07.11.24	Animal ethics and hunting	Prof. Dr. Rudolf Winkelmayer (Retired public and practicing veterinarian, active in the fields of animal welfare and ethics, Pachfurth, Austria)
14.11.24	Foxes and other urban wildlife	Dr. Sophia Kimmig, FU Berlin (AG Ecological Novelty)
21.11.24	Ethical assessment of zoo animals and visits	Prof. Dr. Markus Wild, University of Basel (Professor of Theoretical Philosophy)
28.11.24	Vegan nutrition and ecological footprint	Dr. Anika Döll (GP and nutritionist, Xanten) and Sonja Block (Health for Future, Regensburg)
05.12.24	From farm to home: Animal husbandry between climate change and responsibility *	Dr. Julia Nitsche (Department of Didactics and Educational Research in Health Care) and Prof. Dr. Theresa Busse (Junior Professorship for Digital Health)
12.12.24	God’s work and the lust for meat	Prof. Dr. Anne Käfer, University of Münster (Professor of Systematic Theology)
19.12.24	Insects and other animals live on, in, and around corpses—and we make forensic use of that	Dipl.-Biol. Dr. rer. medic., M.Sc., Ph.D. Mark Benecke, Cologne (Forensic biologist and expert in forensic entomology)
09.01.25	Animal transport and dispatch to third countries	Dr. Alexander Rabitsch, Ferlach (Public veterinarian and specialist staff member at the NGO Animals’ Angels)
16.01.25	Do animals experience placebo effects too? *	Prof. Dr. Jan Ehlers (Head of Department of Didactics and Educational Research in Health Care)
22.01.25	Local film screening: *Die Q ist ein Tier*	In cooperation with Tobby Holzinger Filmproduktion GmbH
23.01.25	BARCAMP “From Knowledge to Action” (*)	Various speakers (see Table 2)
30.01.25	Final Quiz (Kahoot) *	Dr. Julia Nitsche (Department of Didactics and Educational Research in Health Care), Prof. Dr. Theresa Busse (Junior Professorship for Digital Health), Prof. Dr. Jan Ehlers (Head of Department of Didactics and Educational Research in Health Care)

* Sessions conducted by the organizers themselves.

**Table 2:** Barcamp sessions in the winter semester of 2024/2025.

Contents of the Barcamp Sessions	Speaker(s)
Knowledge Protects Animals—Using Social Media	Dr. Karim Montasser (*Der YouTube Tierarzt*)
From Knowledge to Action	Peter Höffken (PETA Germany e.V.)
Animal Sponsorships	Thorsten Emberger (FrecherFuchs)
Quo vadis, Church?	Nicolas Thun (Will-Kirche-Tierschutz.de)
Reflection on “Die Q ist ein Tier” *	Dr. Julia Nitsche (Department of Didactics and Educational Research in Health Care), Prof. Dr. Theresa Busse (Junior Professorship for Digital Health), Prof. Dr. Jan Ehlers (Head of Department of Didactics and Educational Research in Health Care)
Animals in Games—Friend or Foe?	Luisa Hasenack and Lina Andresesn (Medical students, Witten/Herdecke University)
Investigative Journalism in Animal Rights	Scarlett Treml (Animal Rights Watch e.V.—ARIWA)
Who Knows What—Transparency and Education on Animal Testing	Dr. Karl-Gunther Glowalla (University Hospital Jena)
City Pigeons—the Quiet Sufferers	Antonia Grünewald (Animal Shelter Mainz)
Nutrition and Climate Protection	Sonja Schmalen and Antonia Peiler (Health for Future)

* Sessions conducted by the organizers themselves.

**Table 3:** Locations and dates of in-person film screening of “Die Q ist ein Tier”.

Date	Location	City
16.01.25	Bürgerhaus Salzmannbau über Düsseldorf-Vegan.de	Düsseldorf
21.01.25	Kulturbahnhof	Hersbruck
22.01.25	Berlin, Kino in der Brotfabrik	Berlin
22.01.25	Berlin, ProVeg Incubator	Berlin
22.01.25	3001 Kino	Hamburg
22.01.25	Filmhaus	Nürnberg
22.01.25	Universität/EW Gebäude (Raum E10)	Osnabrück
22.01.25	Universum Kino Soest über Ortsgruppe ProVeg Soest	Soest
22.01.25	Filmcasino	Wien (A)
22.01.25	UW/H	Witten
24.01.25	Parkclub	Fürstenwalde
24.01.25	HNEE	Eberswalde
26.01.25	Kräuterpension am Wald (ProVeg-Brunch)	Hanau
22–25.01.25	Filmgalerie	Regensburg

**Table 4:** Study Program Distribution of Participants (Information taken from the details provided by participants during registration).

Field of Study	Number of Participants
Human medicine	242
Dentistry	107
Psychology	86
Veterinary medicine	24
Social sciences/Education	17
Business & Economics	16
Natural sciences	12

**Table 5:** Geographic Distribution of Participants (Information taken from the details provided by participants during registration).

City	Number of Participants
Witten	361
Berlin	85
Bochum	48
Dortmund	37
Köln	32
Leipzig	31
Hamburg	29
München	28
Essen	23
Hagen	17

**Table 6:** Number of participants and interaction data per session in the winter semester 2024/2025.

Date	Participants	Chat Messages ^1^	Q&A Messages ^2^	Number of Polls	Poll Responses
10.10. 24	446	811	22	5	1515
17.10.24	529	245	19	0	0
24.10.24	529	205	13	0	0
31.10.24	480	210	14	6	1644
07.11.24	548	223	29	0	0
14.11.24	518	216	5	1	242
21.11.24	496	174	8	1	246
28.11.24	509	181	8	0	0
05.12.24	474	203	7	6	1195
12.12.24	478	71	14	0	0
19.12.24	451	120	19	0	0
09.01.25	482	91	17	1	188
16.01.25	419	128	3	2	349
23.01.25 ^3^	299	134	0	2	414
30.01.25	399	139	7	0	0

^1^ Messages from participants to all other attendees and to speakers/organizers; ^2^ Messages from participants to the speakers; ^3^ Barcamp with Breakout-Session.

**Table 7:** Number of YouTube Views and Submitted Learning Journals per Session in Winter Semester 2024/2025 (as of 22 May 2025).

Date	Youtube Views	Learning Journals
10.10.24	676	32
17.10.24	410	30
24.10.24	0 *	21
31.10.24	211	25
07.10.24	588	24
14.11.24	181	16
24.11.24	367	32
28.11.24	274	24
05.11.24	131	14
12.12.24	181	6
19.12.24	315	12
09.12.25	223	15
16.01.25	226	8
23.01.25	0	2
30.01.25	0	0
**Total**		**291**

* no permission for recording granted by the speaker.

## Data Availability

The original contributions presented in this study are included in the article. Further inquiries can be directed to the corresponding author(s).
